# Exercise after You Eat: Hitting the Postprandial Glucose Target

**DOI:** 10.3389/fendo.2017.00228

**Published:** 2017-09-19

**Authors:** Melissa L. Erickson, Nathan T. Jenkins, Kevin K. McCully

**Affiliations:** ^1^Department of Pathobiology, Cleveland Clinic Foundation, Cleveland, OH, United States; ^2^Department of Kinesiology, University of Georgia, Athens, GA, United States

**Keywords:** postmeal exercise, postprandial glucose, type 2 diabetes, continuous glucose monitoring, glycemic control

## Abstract

We discuss a novel hypothesis: the effect size of postmeal exercise for attenuating postprandial glucose will be a function of the exercise bout vs. the size of the postprandial glucose response, specifically peak and duration of the postprandial glucose excursion.

## Introduction

Hyperglycemia is a hallmark feature of type 2 diabetes. Sustained high glucose concentrations play a central role in the development of diabetes-related complications (1). Importantly, restoration of glycemic control reduces cardiovascular disease (2). Thus, the primary goal of type 2 diabetes treatment is to achieve and maintain glycemic control. While various therapeutic options are available, glycemic control remains challenging. For example, the long-term performance of hypoglycemic agents is unsatisfactory (2–4).

Traditional markers for glycemic control include fasting glucose, hemoglobin A_1C_ (HbA_1C_), and postprandial glucose. The gold standard for assessing glycemic control is HbA_1C_, which represents a 3-month average of glucose exposure. Postprandial glucose is gaining more recognition as a key glycemic target for therapeutic intervention, as multiple lines of evidence support its use as a clinical marker. Epidemiological studies have shown that postprandial glucose is a better cardiovascular disease predictor than HbA_1C_ (5–7), as well as fasting glucose (8). In addition, interventional studies have shown that reducing postprandial glucose improves glycemic control (9) and leads to reductions in cardiovascular disease risk in people with type 2 diabetes (10).

Due to the growing body of evidence supporting the link between postprandial glucose and cardiovascular disease, the International Diabetes Federation (IDF) published guidelines for postmeal glucose management. Specifically, the target glucose value 1–2 h after meal ingestion is 160 mg/dL (9.0 mmol/L) (11). Recommendations for treatment of postprandial glucose include pharmacologic and non-pharmacological strategies (11). Interestingly, exercise was not described as a treatment option in the IDF recommendation.

Exercise has been shown to be important for both prevention and treatment of type 2 diabetes, and the American Diabetes Association and American College of Sports Medicine have developed exercise guidelines for people with type 2 diabetes (12). However, these guidelines are not specific to postprandial glucose, and they do not mention exercise timing in relation to meal ingestion. The lack of attention to exercise timing in treatment guidelines highlights a need for more research on postmeal exercise and its effects on diabetes-related outcomes.

**Figure 1:**
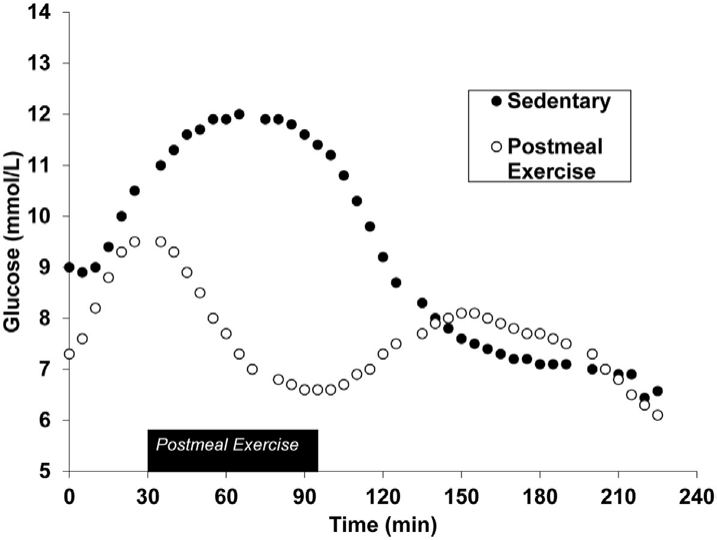
Continuous glucose monitoring data during the postprandial phase of both sedentary and postmeal exercise conditions after a standardized meal in the same individual. Figure has been adapted from previously published works (20). American Physiological Society, permissions for reuse not required due to original authorship.

Postprandial exercise has been shown to be safe (13) and effective in people with type 2 diabetes (Figure 1) (14). Exercise acutely increases glucose uptake in skeletal muscle. This occurs through an insulin-independent process (15, 16) and, therefore, is applicable to type 2 diabetes. Muscle contraction serves as a signal for GLUT-4 receptor translocation on the skeletal muscle plasma membrane (17). GLUT-4 receptors are responsible for transporting glucose from systemic circulation and into skeletal muscle. This effect occurs after just a single bout of exercise, meaning the glucose-lowering effects can be realized immediately (17). Furthermore, the acute nature of this response indicates that long-term training adaptations are not necessary for beneficial effects on blood glucose to occur. The optimal timing for postmeal exercise has been suggested to be 30 min after the start of a meal (18). This is because peak postmeal glucose values typically occur within 90 min (19), and initiating exercise during this time window will blunt peak glucose excursions protecting the endothelial wall from pro-atherogenic glucose concentrations.

## Effective Exercise Considers Glucose Levels

**Figure 2:**
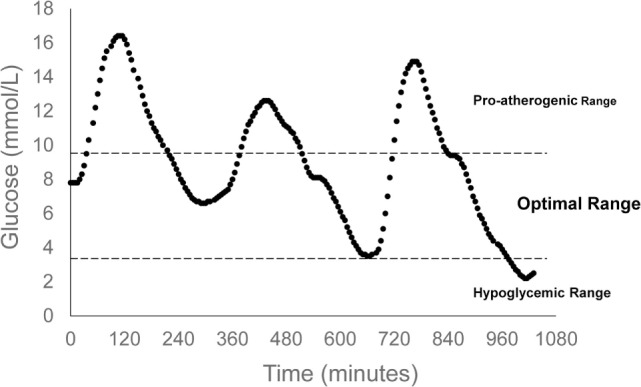
Indicates optimal range of postprandial glucose control. The upper glucose bound is set by the International Diabetes Federation Guidelines, while the lower glucose bound is defined by hypoglycemic risk. Continuous glucose monitoring data are representative of a 24-h glucose profile of an individual with type 2 diabetes. Summary data have been published previously (23).

A key characteristic of type 2 diabetes is an exaggerated glucose response to a meal, and studies using continuous glucose monitoring (CGM) have shown that glucose excursions in people with type 2 diabetes are well above those of non-diabetic controls, even when treated with hypoglycemic agents (21, 22). An optimal postprandial glucose treatment will produce a glucose response that mimics normal glucose tolerance. Measurable parameters of the postprandial glucose response that can be used for interventional guidance include glucose peak and duration of elevation. Those with normal glucose tolerance do not exceed 140 mg/dL and glucose levels return to preprandial levels after 2 h. Given these parameters, postmeal exercise can be strategically applied to lower peak glucose as well as time of elevation, thus resembling normal glycemic control. Effective exercise for type 2 diabetes requires balance, in that, clinically meaningful glucose reductions should be pursued, while minimizing risk for hypoglycemia (Figure 2).

**Figure 3:**
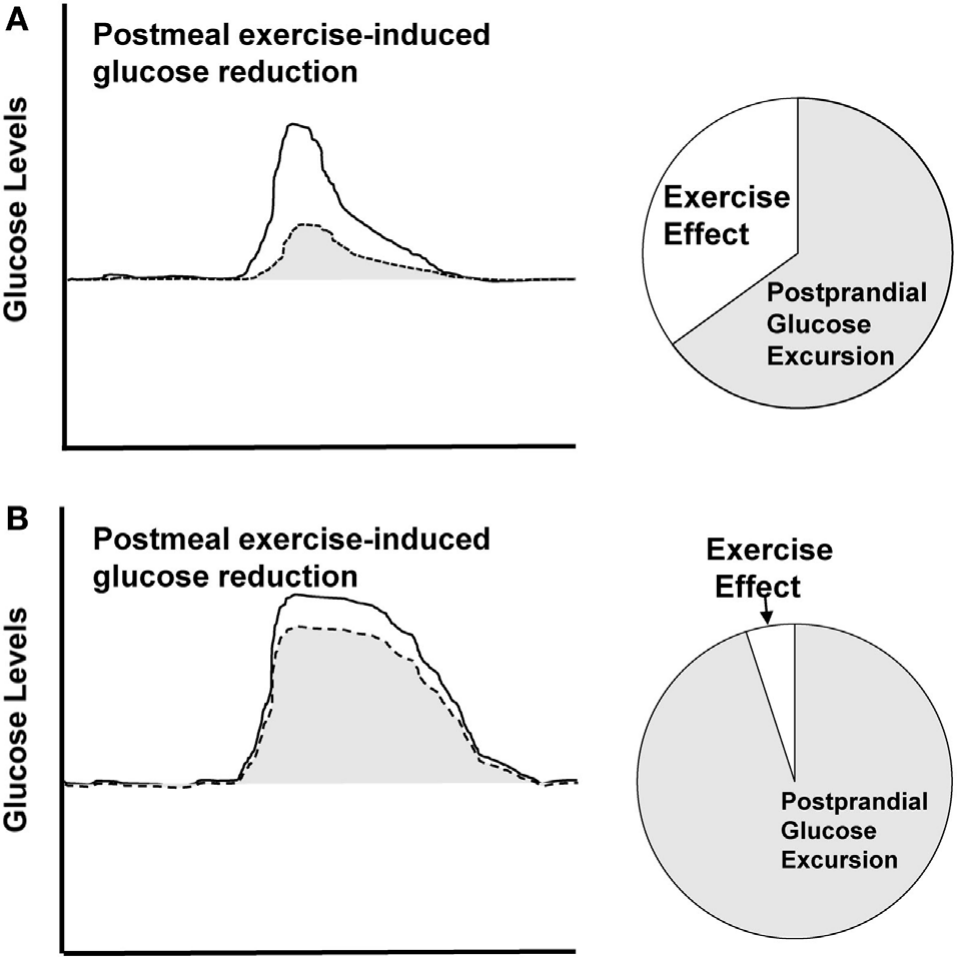
Theoretical depiction. Solid line represents sedentary condition and dashed line represents postmeal exercise condition. **(A)** Displays larger effect size for postmeal exercise-induced glucose reduction in a smaller, shorter excursion. **(B)** Displays smaller effect size for a higher, longer glucose excursion.

We propose that the effectiveness of exercise will be dependent on the postprandial glucose response, including the glucose peak and duration of elevation. Larger amplitude, longer duration glucose excursions will require more intervention than smaller, shorter glucose excursions to produce a normoglycemic pattern. Therefore, the measurable effect size of an exercise bout will be dependent on the glycemic excursion itself, in that, a higher and longer excursion will experience less reduction from the same exercise bout compared to a smaller and shorter excursion (Figure 3). This concept is supported by quantitative comparisons of our previous work. In two distinct studies, we used CGM to assess the effects of postmeal exercise on postprandial glucose excursions in those treated with metformin (20), as well as those treated with more advanced T2D requiring metformin plus additional hypoglycemic agents (23).

We observed different effect sizes for postmeal exercise-induced reductions in 2-h glucose peak, including a large effect size (0.81) in individuals treated with only metformin (20) vs. a moderate effect size (0.56) in those treated with additional hypoglycemic agents (23). We propose that these differential results can be explained by two key differences among studies, including (1) differences in the applied exercise bout, as well as (2) differences in the amplitude of the postprandial glucose peak. The larger effect size was observed in the study that applied the larger exercise stimulus, i.e., exercise that was longer in duration (50 vs. 30 min) and modestly higher in intensity (60% VO_2_ max vs. 50% VO_2_ max). In addition, the amplitude of the 2-h glucose peak measured in the control condition was lower in the metformin study participants compared to that of the metformin plus add-on hypoglycemic agent study participants (12.0 vs. 14.5 mmol/L, respectively), indicative of an easier glucose “target” for reduction. This comparison suggests that a larger exercise stimulus (longer in duration, higher in intensity) applied to the smaller glucose peak resulted in a more effective strategy for glucose attenuation. One interpretation of this comparison is that the effectiveness of a postmeal exercise bout is a function of the amount of exercise vs. the size of the glucose peak.

To further explore this concept, a second comparison of the two studies was completed. The aim of this analysis was to use CGM data to quantify the amount of time exercising vs. the amount of time spent in postprandial hyperglycemia. This can be thought of as the percentage of time in which the glucose excursion was being intervened upon by exercise. This analysis revealed differences among our two studies. The percentage of time that was “treated” by exercise was 34% in the metformin study (20), vs. only 16% in the add-on therapy (23). These findings are consistent with the measured effect sizes, in that the larger effect size corresponded with the larger percentage of treated time (ES: 0.81; 34% of time was “treated” with exercise) and the smaller effect size corresponded with the small percentage of treated time (ES: 0.56; 16% of time was “treated” with exercise). These findings further support the concept that the effectiveness of exercise will be a function of the exercise bout vs. the size of the postprandial glucose response.

Significant glucose reductions have been reported in people with type 2 diabetes using a large variety of exercise strategies. This includes continuous (24, 25) and interval protocols (20, 23). In addition, various durations (20–60 min) and intensities have been shown to be effective (26, 27). Furthermore, multiple modes of exercise have been used including walking and cycling (24, 25). More recently, high-intensity interval training has been shown to be a promising approach for improving health outcomes in the people with type 2 diabetes (28–30). Taken together, these studies show there are numerous strategies to prescribe exercise. It is currently not clear if one variable is more important than another for postprandial glucose control. However, in the case of postmeal exercise approaches, it seems evident that maximizing the glucose-lowering power of an exercise bout will require taking the size of the glucose excursion itself, into account.

## Complementary Effects of Drugs and Exercise

Hypoglycemic agents are a mainstay in type 2 diabetes treatment. Thus, the combined effects of hypoglycemic agents and exercise should remain a high priority for future investigations. Metformin is the first-line therapy (31) for the prevention and treatment of type 2 diabetes. During disease progression, a variety of hypoglycemic agents can be used for glycemic control. Currently, there are nine available FDA-approved classes of oral hypoglycemic agents (32) and several injectable agents. Some drug classes specifically target and reduce postprandial glucose, including α-glucosidase inhibitors, DPP-4 inhibitors, glinides, GLP-1 derivatives, short-acting sulfonylureas, and insulin regimens (31). All of these medications are recommended to be taken along with regular exercise.

The target tissues and mechanisms of action widely vary among drug classes. Subsequently, these drug classes have differing effects on the 24-h glycemic profile. Some drugs effectively lower fasting glucose, while others target postprandial glucose. Postmeal exercise may be an effective complement to these agents. In fact, the combination of postmeal exercise and hypoglycemic agents has been shown to produce further glucose-lowering effects compared to drug treatment alone (20, 23). Additional experimental studies are needed to determine the interactive effects of postmeal exercise among various drug classes. This will involve appropriately timing medication and exercise in order to avoid hypoglycemia.

Insulin and insulin-analog regimens have been specifically designed to reduce postprandial glucose. Incorporation of postmeal exercise alongside insulin therapy may also have beneficial health effects. If postmeal exercise is effective enough, it could potentially lead to a reduction in insulin dose. A study in participants with type 1 diabetes found that prolonged walking (~40–50 km) led to profound reductions in insulin administration (26%) compared to a sedentary day (33). Future studies should investigate the effectiveness of more feasible exercise strategies, including postmeal exercise, as a complementary treatment to insulin.

For safety reasons, optimal diabetes treatments should have a low probability for eliciting hypoglycemia. The counter-regulatory response is a natural physiological process that defends against hypoglycemia, and this can occur if glucose falls too low during exercise. The counter-regulatory release of hormones into the circulation, including glucagon, catecholamines (epinephrine and norepinephrine), cortisol, and growth hormone is triggered when glucose drops below 3.8 mmol/L (34). This effect has been demonstrated experimentally in people with type 2 diabetes (25, 35) and should remain a consideration with prescribing exercise alongside hypoglycemic agents.

## Glucose-Guided Approach

Our current hypothesis that the effectiveness of an exercise bout will be dependent on the size of the glucose excursion itself. Therefore, optimal exercise approaches will require knowledge of glucose values in real time. One commonly used approach is self-monitoring capillary glucose with finger sticks and glucometers. When timed correctly, this method can be used to assess acute fluctuations in glucose after meals. In addition, CGM will likely play an important role. CGM technology uses a small probe within the subcutaneous tissue that samples and measures glucose concentrations in the interstitial fluid. Future studies should determine the most effective and feasible approach for glucose-guided exercise prescriptions, which may involve a hybrid approach of glucometers and CGM.

## Conclusion

Improving the treatment of type 2 diabetes is a major health care need. Taming postprandial glucose excursions can be accomplished by exercising after meals. The effectiveness of an exercise bout for lowering glucose will be dependent upon the size (peak and duration) of the postprandial glucose excursion. Larger excursions necessitate more aggressive intervention, while smaller excursions are easier targets for attenuation. Glucose monitoring techniques, such as glucometers and CGM technology, may have an important role in quantifying the effectiveness of exercise bouts.

## Author Contributions

ME, NJ, and KM developed ideas. ME drafted manuscript. NJ and KM edited and approved manuscript.

## Conflict of Interest Statement

The authors declare that the research was conducted in the absence of any commercial or financial relationships that could be construed as a potential conflict of interest.
